# Novel Intraocular Therapy in Non-infectious Uveitis of the Posterior Segment of the Eye

**Published:** 2013

**Authors:** Michael Mikhail, Ahmed Sallam

**Affiliations:** 1 Royal Victoria Hospital, Belfast, UK,; 2 Gloucestershire Hospitals NHS Foundation Trust, UK

**Keywords:** Non-infectious Uveitis, Posterior Segment, Intraocular Therapy, Retisert, Iluvien, Ozurdex, Methotrexate, sirolimus

## Abstract

This article reviews the new clinically relevant data regarding the intraocular treatment of non-infectious uveitis. Triamcinolone acetonide is the most commonly used intravitreal corticosteroid for treatment of uveitis and uveitic macular oedema. The drug is available at low cost but it is associated with a high risk of raised intraocular pressure (IOP) and cataract and is not licensed for intraocular use. Dexamethasone implant (Ozurdex®) appears to have a better safety profile, and a slightly long-lasting effect than triamcinolone acetonide. Fluocinolone acetonide implant (Retisert**®**) implant allows the release of corticosteroids at a constant rate over a 3-year period, but it requires surgical placement and its use is associated with a very high risk of cataracts and raised intraocular pressure. Iluvien**®** is another fluocinolone acetonide implant that could represent a more convenient treatment option for such cases in the future as it can be inserted into the vitreous cavity through 25-gauge injector system in an outpatient setting. To circumvent the risks associated with corticosteroids use, non-corticosteroids related therapeutics including intravitreal methotrexate; anti-vascular endothelial growth factor treatments and intravitreal sirolimus have been recently developed.

## INTRODUCTION

Uveitis belongs to a group of intraocular inflammatory disorders affecting the uvea, which can cause significant visual impairment and may result in partial or complete loss of vision. It encompasses a wide range of clinical phenotypes, and can be classified anatomically into anterior, intermediate, posterior and panuveitis (1).

 Uveitis can also be divided based on its aetiology into infectious, non-infectious, and masquerade syndromes (neoplastic and drug-induced). The course of uveitis may be defined as acute, recurrent or chronic (1). It is estimated that non-infectious uveitis involving the posterior segment of the eye affects around 3 to 10 persons per 100,000 in the European Union (1,500 and 5,000 cases per year in England). The true incidence is difficult to determine as some cases may resolve spontaneously and not present clinically (1,2).

 Uveitis affecting the posterior segment of the eye is often unresponsive to topical administration of steroids due to less than optimum therapeutic drug penetration beyond the lens. Periocular and subtenon steroids could be effective in treating some patients with uveitis associated cystoid macular edema (CME) but their successful use has been limited to mild cases due to poor absorption of the drug when delivered through this route intraocularly (3). Long-term systemic corticosteroid therapy is required in patients with an associated systemic disease and in those with bilateral ocular inflammation. Although effective, it is associated with a variety of potentially serious adverse effects such as induction or worsening of hypertension and diabetes mellitus, osteoporosis, and adrenal suppression. Second line immunosuppressive drugs and biological agents such as tumour necrosis factor alpha inhibitors are used as ‘steroid sparing’ treatments, however, they have their own systemic risks, which are likely to limit their clinical use.

 Intravitreal drug delivery allows rapid and high concentrations of the drug into the eye as it bypasses the blood ocular barriers and at the same time is associated with the lowest incidence of drug related systemic toxicity (3). However, its significant drawbacks include the possibility of retinal toxicity and mechanical injury to intraocular structures like the crystalline lens or retina. Intraocular injections are also associated with a small risk of endophthalmitis. 

 This article reviews the current literature on the use of intraocular drugs for treatment of non-infectious uveitis affecting the posterior segment of the eye. Triamcinolone acetonide is currently the most commonly used intravitreal treatment. However, despite its potency, it has a short therapeutic duration as well as a significant adverse effect profile in terms of cataract formation and increase in IOP. Recently, several slow release corticosteroid implants have been developed in order to prolong the effectiveness of the drug. New non-corticosteroid related therapeutics; including intravitreal methotrexate, anti-vascular endothelial growth factor treatment and intravitreal sirolimus to treat intraocular treatment of non-infectious uveitis have also been developed to avoid the ocular side effects inherent to the use of intraocular steroids.

## Intravitreal triamcinolone acetonide

The use of intravitreal triamcinolone acetonide (IVTA) at a concentration of 2-4mg in 0.1ml is currently a common practice for the treatment of uveitis affecting the posterior segment of the eye (4-6). The Federal Drugs Administration (FDA) in the USA has approved two formulations of triamcinolone acetonide for intraocular use. Triamcinolone acetonide is not licensed for intraocular use in the European Union but is routinely employed for the treatment of non-infectious posterior uveitis and uveitic CME. The typical duration of the treatment is 4-5 months, with the maximum effect on vision occurring within six weeks (4). A study of the pharmacokinetics of the drug following an injection of 0.1 ml (0.3mg) of triamcinolone acetonide in 42 vitrectomised eyes and 42 non-vitrectomised eyes showed that IVTA decreases more rapidly in the vitrectomised eye than in the non-vitrectomised eye (5).

Kok *et al*., (6) studied the short-term outcome of intravitreal triamcinolone acetate in the treatment of uveitic CME. This study is the largest of its kind and involved 65 eyes of 54 patients. All had uveitis related CME which was unresponsive to treatment combinations of oral corticosteroid, periocular orbital floor corticosteroid injections, and second-line immunosuppressive agents. Over a mean follow-up of 8 months, 83% of eyes responded to a 4mg dose of IVTA of which 51% gained at least two Snellen lines. The improvement in VA was more significant if the duration of CME before IVTA was 12 months and if patients were 60 years old. This treatment protocol also enabled the doses of oral corticosteroids and second line immunosuppressive agents to be reduced (6).

 As mentioned before, the side effects of steroids significantly limit their use. The most common adverse side effects are raised IOP and cataract. Raised intraocular pressure is seen in 29%-50% of patients within a year (7). In most cases, this is transient and can be controlled medically, but there have been a few reports of patients requiring surgical intervention (8). Previous studies reported the rate of cataract development at 15-30% after one injection (9).

## Dexamethasone (Ozurdex®) intravitreal implant

Ozurdex® (Allergan Pharmaceuticals) is a biodegradable dexamethasone intravitreal implant that contains 0.7mg of dexamethasone. Ozurdex® is placed intravitreally through the pars plana with an injector using a 22-gauge needle device. The insert provides peak doses for an initial 2 months followed by lower doses for up to 6 months and can be safely performed as an outpatient procedure. Ozurdex® received FDA approval in June 2009 for the treatment of macular edema associated with retinal vein occlusion, and for the treatment of non-infectious posterior uveitis in September 2010.

 The clinical efficacy of Ozurdex® in the treatment of non-infectious posterior uveitis has been assessed in a single study; a phase III, multicentre, randomised, double-masked, sham-controlled trial known as the HURON study (chronic uveitis evaluation of the intravitreal dexamethasone implant) (10). The 26-week, prospective, multicenter, masked study included 229 patients with intermediate or posterior uveitis. Participants were randomized to receive a single treatment with a 0.7-mg dexamethasone implant (n=77), a 0.35-mg dexamethasone implant (n=76), or a sham procedure (n=76). Eighty-one percent of patients had intermediate uveitis. At the eighth week primary endpoint, 47%, 36% and 12% of patients had no vitreous inflammation, respectively. The response was maintained at week 26. In addition, both treatment groups achieved a 3-line improvement in visual acuity and reduced central macular thicknesses on optical coherence tomography (OCT) at 8 weeks that was statistically significant compared to the sham group. Regarding the complication rate, the incidence of cataract reported in phakic eyes was nine of 62 (15%) in the 0.7-mg implant group. Twenty-three percent of eyes in the 0.7 mg Ozurdex® group required IOP-lowering agents but none needed surgical intervention for glaucoma. The effect of repeat injections on cataract and IOP remains unknown.

## Fluocinolone acetonide (Retisert®) implant

The Retisert**® **intravitreal implant (Bausch & Lomb, Rochester, New York, USA) is a slow release corticosteroid implant that contains fluocinolone acetonide and releases the drug at a constant rate over a three year period of time. In chronic uveitis, cumulative structural damage and loss of vision result from recurrent episodes of inflammation (11). The goal of uveitis treatment should not only be to suppress inflammation when it occurs but also to attain complete remission of inflammation in the longer term. Fluocinolone is a corticosteroid with a high potency, low solubility and a very short duration of action in the systemic circulation (12). Implantation of this device requires surgery with the implant being placed through a pars plana incision and anchored to the sclera with sutures (13,14).

 The efficacy of fluocinolone implant has been evaluated in several studies (15-17). In one of these studies, a total of 278 patients with recurrent non-infectious posterior uveitis were randomized to receive a 0.59-mg (n = 110) or 2.1-mg (n = 168) implant. Results showed that the Retisert**®** reduced the rate of recurrences from 51.4% in the 34 weeks preceding implantation to 6.1% post-implantation in the study eyes. Comparatively, there was a significant increase in the recurrence rate in the fellow non-implanted eyes from 20.3% pre-implantation to 42.0% post-implantation. Visual acuity was stabilized or improved in 87% of implanted eyes. The percentage of eyes that required systemic medications, periocular injections, and topical corticosteroids decreased from 52.9%, 63.0%, and 35.7%, pre-implantation to 12.1%, 2.2%, and 16.5% post-implantation, respectively.

 In terms of complications, cataract surgery was required in 93% of the cases and 51.1% of the eyes receiving the implant required antihypertensive drops, with 5.8% undergoing glaucoma filtering procedures (16). Other reported side effects include hypotony (18), implant malfunction (19), retinal detachment, endophthalmitis, scleral thinning (18), and the development of opportunistic intraocular infections including herpetic retinitis (20-21).

 Further evidence to support the use of fluocinolone acetonide implant in vision threatening non infectious uveitis stems from The Multicenter Uveitis Steroid Treatment (MUST) trial (22), a randomized controlled clinical trial comparing local therapy with fluocinolone acetonide intraocular implant standard therapy with systemic corticosteroid therapy supplemented, when indicated, by corticosteroid-sparing therapies. Study results (23) indicated that in each treatment group, mean visual acuity improved over 24 months, with neither approach being demonstrably superior to the other. Therefore, the specific advantages and disadvantages associated with each treatment should dictate which of the two treatments should be selected, which would likely be a consideration of individual patients' particular circumstances.

## Fluocinolone acetonide (Iluvien®) implant

Iluvien**®** (Alimera Sciences Inc.) is another fluocinolone acetonide intravitreal insert, which is designed to deliver corticosteroid to the retina for up to 3 years (24). It is injected through a 25-gauge injector system in an outpatient setting. Although, it uses same drug matrix as Retisert**®**, it releases the drug at a lower dose (0.2 or 0.5ug/day versus 0.59ug/day with Retisert**®**) (23). There is no published evidence for its effect in treating uveitis; however, positive effect on diabetic macular oedema was shown on a large scale previous study (25).

## Antivascular endothelial growth factor agents

The pathophysiology of CME associated with uveitis is not completely understood. Chronic intraocular inflammation is associated with increased production of inflammatory mediators, including vascular endothelial growth factor (VEGF), which are hypothesized to disrupt the blood-retinal barrier on the endothelium of retinal vessels, resulting in subsequent macular edema. A study in human eyes with uveitis and CME showed increased concentration of VEGF in the aqueous humor (26). It is suspected to play a role in the loss of vascular integrity in the eye and is known to be induced by inflammatory cytokines, such as interleukin 1 and interleukin 6, which have been found to be elevated intraocularly in uveitis patients (27,28). Therefore, inhibition of inappropriate VEGF activity is a potential new approach to treatment of CME in this population. 

 A prospective, non-comparative, interventional case series (29) looking at seven consecutive patients with controlled uveitis and refractory CME who had little to no results from corticosteroid treatment were studied by Nisha and associates. At 3 months, the mean increase in visual acuity for the 6 patients who completed follow-up was 13 letters (2.5 lines). Both VA and central retinal thickness improved significantly between baseline and 3 months demonstrating that intravitreal ranibizumab could result in regression of uveitis-associated CME in patients who are refractory to or intolerant of systemic corticosteroid therapy. 

 Several studies (30-32) have also compared the use of intravitreal bevacizumab to IVTA in the treatment of uveitic macular oedema. They all concluded that there were better visual improvement and decreased macular thickness in the IVTA treated eyes. 

 Anti-VEGF agents have the advantage over various corticosteroids since they are much less likely to cause cataract progression or a rise in IOP. However, they have less of an anti-inflammatory effect, making them less suitable for the treatment of severe CME that is primarily driven by inflammation (33). Nevertheless, anti-VEGF agents do have a pivotal role in the treatment of inflammatory choroidal neovascular membranes (CNV). Adan *et al*., (34) assessed the effects of intravitreal bevacizumab injection as primary treatment of inflammatory CNV. The study demonstrated complete resolution of CNV in 100% of eyes. Of a total of nine eyes that were followed for seven months, visual improvement occurred in eight eyes and stabilized in one eye. No patient had visual deterioration and a mean 1.3 injections/eye were needed. Chan *et al*., (35) reported visual and anatomic improvements in eyes with idiopathic CNV and CNV attributable to punctate inner choroidopathy (PIC) and central serous choroidopathy. All 15 eyes had improvement in VA with a mean improvement of 2.9 lines at six months, as well as a reduction in central foveal thickness on OCT. Further studies are warranted to define its exact role as a monotherapy for eyes with uveitic complications and the potential for combination treatment with corticosteroids. Finally, it is important to mention that use of anti-VEGF agents could be associated with risk of cardiovascular events, though this is debatable (36).

## Intravitreal Sirolimus

Sirolimus is a macrolide antibiotic also known as rapamycin. It has broad immunosuppressive and anti-proliferative properties (37-39). Sirolimus arrests cell cycle progression by direct interaction with aimmunophilin FK binding protein 12 (FKBP-12) resulting in sirolimus-FKBP-12 complex which then binds to and inhibits mammalian target of rapamycin (mTOR). The inhibition of mTOR blocks IL-2 mediated signal transduction pathways that prevent cell cycle progression (37-39).

 Sirolimus as a Therapeutic Approach for Uveitis (SAVE) (40) is a prospective, randomized, open-label, phase I study that evaluated the ocular tolerability and efficacy of Sirolimus administered as subconjunctival or intravitreal injections in patients with non-infectious uveitis. Thirty patients were enrolled and randomized in 1:1 ratio to receive either intravitreal or subconjunctival sirolimus injections at days 0, 60, and 120. Primary endpoint was at month 6 and all subjects with active uveitis at baseline showed reduction in vitreous haze. Changes in the inflammatory indices were statistically significant (p < 0.05) in both study groups. Thirty percent of patients gained one or more lines of visual acuity, 20% lost one or more lines, and 50% maintained the same visual acuity. No serious adverse events related to the study drug were demonstrated in this study. 

 Intravitreal sirolimus is currently being evaluated in a phase III study named SAKURA, which will assess the safety and efficacy of 3 different doses of this drug in non-infectious uveitis affecting the posterior segment of the eye. This study involves multiple centers from Europe, India, North America as well as the Middle East and has just finished recruiting.

## Anti-Tumor necrosis factor alpha

Tumor necrosis factor alpha (TNF-α) is a pro-inflammatory cytokine produced by macrophages and T-cells. It plays an important role both in inflammation and apoptosis. In the eye, TNF-α appears to have a role in the pathogenesis of inflammatory, edematous, neovascular and neurodegenerative disorders (41-43).

Drugs that neutralize TNF-α, i.e. TNF-α blockers are expected to have a positive effect on reducing symptoms of various diseases associated with increased TNF-α activity (41-43).

 Because of their demonstrated systemic benefit, there has been a recent interest in targeted intraocular treatment with anti-TNF-α drugs (41-43). So far, 2 anti-TNF drugs have been used intravitreally and these are infliximab, a chimeric monoclonal antibody composed of human constant and murine variable regions, and adalimumab, a humanized monoclonal antibody. However, the available data on their intravitreal use is limited and does not appear to be promising. Infliximab, while shown to be effective in reducing macular oedema and vitritis, is associated with a significant risk of severe panuveitis and vitreous opacification (44). This has led to a call for a moratorium on the clinical use of intravitreal infliximab outside well-monitored trials (45). Tsilimbaris and coworkers (46) investigated the safety of injecting adalimumab into the vitreous of in rabbits. Their data showed that intravitreal concentrations of up to 5 mg were not associated with any retinal toxicity. Clinical use of intravitreal adalimumab in uveitic CME also demonstrated no safety concerns; however, it failed to produce any significant improvement in vision or macular thickness (47).

## Methotrexate

Methotrexate inhibits tetrahydrofolate synthesis by inhibiting the enzyme dihydrofolate reductase. Folic acid is important for the synthesis of DNA, RNA, and proteins. Methotrexate reduces B and T cell proliferation, and is commonly used either orally or subcutaneously at a dosage of 15–25 mg weekly in adult patients with uveitis (48). 

 Methotrexate is also effective for intraocular lymphoma when used intravitreally (49). This mode of delivery has more recently been tried in posterior segment uveitis however this was on a small scale (50). In a pilot study, it was found to be effective in reducing vitritis and macular oedema without raising IOP in patients with a history of steroid response. The onset of effect was within 1 week and lasted 4 months with no statistical difference found between the best visual acuity obtained after methotrexate injection and previous use of IVTA (51).

## CONCLUSION

The goal of uveitis treatment is not only to suppress inflammation when it recurs but also to attain complete remission of inflammation, and thus prevent complications and permanent cumulative retinal damage (11). Therefore, long-term control of inflammation is crucial if vision to be preserved. 

 Corticosteroids remain the mainstay of intraocular treatment of uveitis and uveitic CME, but their use has significant adverse effects in terms of cataract and raised IOP. Although Triamcinolone, the most commonly used intraocular steroid, is available at a low cost, Ozurdex® dexamethasone implant appears to have less adverse effects and lasts longer. Fluocinolone implants last up to two and a half years, which is an advantage in patients with chronic uveitis. However, their use is associated with a very high risk of cataract, and a significant percentage of patients require IOP reduction surgery. Retisert**®** is the one currently available for uveitis but its insertion requires surgery. As Iluvien**®** could be injected into the vitreous cavity through a minimally invasive procedure in an outpatient setting; it could in principle represent a more convenient treatment option for treating non-infectious uveitis in the future. 

 To circumvent the risks associated with corticosteroids use, several non-corticosteroid novel therapeutics have so far been explored such as intravitreal methotrexate, anti-VEGF, anti-TNF α and sirolimus with varying success. Methotrexate offers a better alternative to corticosteroid treatment than anti-VEGF agents; however, the published data supporting the use of intravitreal methotrexate in the treatment of uveitis is still limited and is based on small case series (51). Controlled trials would be required to confirm these outcomes. While the use of intravitreal anti-TNF α does not appear to be promising, sirolimus delivered as an intravitreal injection has previously demonstrated bioactivity as an immunomodulatory agent in reducing vitreous inflammation and could be a viable option pending the results of the phase III SAKURA study. 
